# Methodology of generation of CFD meshes and 4D shape reconstruction of coronary arteries from patient-specific dynamic CT

**DOI:** 10.1038/s41598-024-52398-5

**Published:** 2024-01-25

**Authors:** Krzysztof Psiuk-Maksymowicz, Damian Borys, Bartlomiej Melka, Maria Gracka, Wojciech P. Adamczyk, Marek Rojczyk, Jaroslaw Wasilewski, Jan Głowacki, Mariusz Kruk, Marcin Nowak, Ziemowit Ostrowski, Ryszard A. Bialecki

**Affiliations:** 1 Department of Systems Biology and Engineering, Silesian University of Technology, 44-100 Gliwice, Poland; 2 Biotechnology Centre, Silesian University of Technology, 44-100 Gliwice, Poland; 3 Biomedical Engineering Lab, Department of Thermal Technology, Silesian University of Technology, 44-100 Gliwice, Poland; 4 Third Department of Cardiology, Faculty of Medical Sciences in Zabrze, Medical University of Silesia, 41-800 Zabrze, Poland; 5 Silesian Centre for Heart Diseases, 41-800 Zabrze, Poland; 6 Department of Radiology and Radiodiagnostics, Medical University of Silesia, 41-800 Zabrze, Poland; 7 Department of Coronary and Structural Heart Diseases, National Institute of Cardiology, 04-628 Warsaw, Poland; 8 Department of Mechanics of Materials and Structures, Gdańsk University of Technology, 80-233 Gdańsk, Poland

**Keywords:** Image processing, Cardiovascular diseases, Fluid dynamics, Biomedical engineering

## Abstract

Due to the difficulties in retrieving both the time-dependent shapes of the vessels and the generation of numerical meshes for such cases, most of the simulations of blood flow in the cardiac arteries use static geometry. The article describes a methodology for generating a sequence of time-dependent 3D shapes based on images of different resolutions and qualities acquired from ECG-gated coronary artery CT angiography. The precision of the shape restoration method has been validated using an independent technique. The original proposed approach also generates for each of the retrieved vessel shapes a numerical mesh of the same topology (connectivity matrix), greatly simplifying the CFD blood flow simulations. This feature is of significant importance in practical CFD simulations, as it gives the possibility of using the mesh-morphing utility, minimizing the computation time and the need of interpolation between boundary meshes at subsequent time instants. The developed technique can be applied to generate numerical meshes in arteries and other organs whose shapes change over time. It is applicable to medical images produced by other than angio-CT modalities.

## Introduction

Epidemiological data show that coronary artery disease affects approximately 200 million people worldwide, causing almost 9 million deaths^1^. In addition to traditional, invasive, and noninvasive diagnostic and therapeutic techniques, methods of computer simulations of blood flow in coronary vessels based on Computational Fluid Dynamics (CFD) are playing an increasingly important role. The use of CFD in coronary artery disease is addressed in several review articles^1–3^, demonstrating the usefulness of this medical technique in clinical practice. Reference^1^ discusses a survey completed by almost 500 interventional cardiologists. Almost 90% of the respondents expressed their willingness to use CFD in their clinical practice.

CFD simulations provide direct information on the temporal and spatial distribution of pressure and flow patterns in the vessels. Many other parameters can be determined in post-processing, including wall shear stress oscillations, a quantity that controls the process of atherosclerotic plaque deposition^4^. The deformed shear stress profile changes the morphology of endothelial cells by activating multiple signal channels^5,6^. Reference^7^ makes the hypothesis that low oscillatory sheer stress promotes plaque deposition due to increased leukocyte adhesion, plasma lipoprotein permeability, smooth muscle cell migration, and reactive oxygen species.

The measure of the oscillatory nature of the tangential stresses on the wall of the vessel is the measure of the shear stress oscillation (OSI) and is defined as^8^:

1 $$\begin{aligned} OSI = 0.5 \times \left( {1 - \frac{{\left| {\mathop \smallint \nolimits _{0}^{T} \overrightarrow{WSS} dt} \right| }}{{\mathop \smallint \nolimits _{0}^{T} \left| {\overrightarrow{WSS} } \right| dt}}} \right) , \end{aligned}$$

where $\overrightarrow{WSS}$ is the tangent stress on the wall of the vessel and T denotes the time.

There are several other indicators of oscillation of the tangential stress field: time-Averaged Wall Shear Stress (AWSS), time-Averaged Wall Shear Stress Vector (AWSSV), Oscillatory Shear Index (OSI), and Relative Residence Time (RRT).

In addition to WSS oscillations, CFD calculations can also predict the composition of the atherosclerotic plaque, its tendency to rupture, and the noninvasive determination of the fractional flow reserve parameter (FFR)^9^. The latter is defined as the ratio of the maximum achievable blood flow through a stenosis to the maximum flow in the same vessel in the hypothetical absence of the blockage. The fractional flow reserve (FFR) is routinely used to determine the severity of the injury prior to annual percutaneous intervention (PCI). CFD simulations are also used to design coronary artery stents^10^.

CFD calculations in the coronary arteries are associated with severe limitations. The main source of difficulty is the mapping of the complex geometry of the vascular tree. The restoration of the time-dependent geometry shape of the Left Anterior Descending artery (LAD) is the main topic of this article. The developed methodology is applicable to any artery whose medical images are available.

The cyclic character of cardiac flow, the presence of branches, the separation and reattachment of the flow, the vasomotorism and the geometry of the variable myocardium within the cardiac cycle contribute to the complexity of the blood flow pattern. All these features require the use of good quality numerical grids, not only with adequate resolution but also applicable to temporal changes of the geometry of computational domains^3^.

This last aspect requires further explanation. To reflect the change in geometry and, thus, the numerical grid in time, advanced CFD programs use the MeshMorphing option^11,12^. The working principle of its operation is to change the numerical grid so that only the positions of the boundary nodes change, while the connections of the nodes into the elements (connectivity matrix, topology) remain unchanged. Due to this, difficult-to-control interpolation errors between grids with different topologies are avoided, which also leads to the acceleration of calculations.

Time-dependent 3D shapes of blood vessels can be obtained using radiological modalities such as computed tomography angiography, nuclear magnetic imaging, intravascular ultrasound, or standard ultrasound spectroscopy. For a given instant in time, the raw medical image is processed using segmentation and smoothing to produce a 3D shape of the vessel. Under assumed boundary conditions, the blood flow pattern and pressure distribution in the vessel can be simulated in the retrieved geometry. This approach does not require knowledge of the difficult-to-determine *in vivo* material properties of adjacent tissues to the vessel wall. It opens the way to virtual therapy, based on patient-specific data.

Due to the difficulties associated with the time-dependent shapes of vessels, blood flow simulations are typically based on a series of static geometries^13,14^. Several attempts were published where lumen changes were accounted for^15^, but the centerline remained constant over time. In^16^ the deformation of the artery was taken into account by extraction of the centerline, but the lumen of the vessel was reconstructed as a circle, while its radius was based only on coarse segmentation. Reference^17^ accounts for changes in the length of the vessel. However, it neglects the variation in the lumen of the arteries. Reference^18^ describes a technique in which the topology of the CFD mesh remains unchanged. The idea is to generate an artificial, simplified shape, being a cluster of 3D geometry primitives, that is similar to the object of interest. Within this simplified shape, the volumetric CFD mesh is generated. This mesh is then transformed into the subsequent shapes of the 4D medical images. The transformation is accomplished by resorting to an inverse with a Tikhonov regularization. A more advanced approach has been described in^19^ where CFD solution has been analyzed in a portion of the right coronary artery without bifurcations. In this paper, the movement of the vessel has been taken into account. The lumen cross-sectional shapes remains unchanged during the cardiac cycle.

Theoretically, it is also possible to simulate blood flow using the fluid-structure interaction; however, this requires knowledge of not only the mechanical properties of the wall but also the deformation of the tissues in contact with the vessel. In the case of coronary arteries attached to the myocardium, this requires movement and deformation of the heart muscle. No trace of such an approach has been found in the literature.

The direct motivation for undertaking the research underlying this paper was to use CFD to investigate hemodynamics in the coronary arteries in the presence of a myocardial bridge (MB).

The coronary arteries deliver blood to the heart muscle. They run along the outer surface of the heart in the epicardium. This location prevents the vessels from compressing in systole. Myocardial bridging is a congenital condition in which heart muscle bands overlay a segment of the coronary artery called the myocardial bridge. The geometry of the MB differs greatly in length, location, and depth. The most common location of MB is the left anterior descending coronary artery, but it can be found in any epicardial artery^20,21^. The prevalence of MB is difficult to assess because it varies greatly depending on the method used to detect this condition, and some cases do not show visible symptoms. Angiography studies give estimates between 0.5 and 12%^22^, while autopsy rates report a frequency of 5 to 86% with a mean of 25%. A detailed review of the prevalence of MB is presented in Ref.^23^.

The presence of MB changes the pattern of blood flow in the coronary arteries, resulting in the deposition of an atherosclerotic plaque at the proximal end of the MB, while the segment under the MB and distal to it is practically free of plaque^21^. Based on 150 autopsied hearts, Ref.^24^ formulates the hypothesis that the reason for this behavior is the low wall shear stress at the proximal end.

The only plausible method to investigate the abnormal distribution of sheer wall stress in the vicinity of the MB is to simulate blood flow. This approach has been used by numerous research teams. However, the dominant approach has been to retrieve the geometry of the coronary arteries using known modalities. First, the raw images are segmented and, in the next step, CFD is applied to simulate blood flow^25–32^. The cited papers differ by the simplifications made in the formulation: 2D and 3D models, steady-state, boundary conditions, etc. All studies assume no change in vessel length or lumen shape, except for the MB itself, for which the temporal changes of the vessel perimeter are predefined.

Our study, using a collection of ECG-gated angio-CT coronary vessel images recorded in subsequent steps of the cardiac cycle. To minimize exposure to X-rays, the resolution of the images was lower than that used separately for high-resolution diastole and systole images. Furthermore, the dose within the cycle is modulated. Variations in X-ray intensity were introduced to minimize the dose absorbed by the patients. As a result, the raw data set consisted of images of various resolutions and quality. The segmentation of the source images has been carried out using the ITK SNAP package^33^. The results were smoothed using (GeoMagic software^34^), and co-registered (ANTs package^35^). A key element of the proposed method is the generation of a CFD mesh of identical topology at each time instant. Thanks to this, the interpolation errors between grids are avoided and the CFD calculation time is reduced.

## Materials and methods

### Patient data

Coronary computed tomography angiography (CCTA) scans were performed using a 128-slice dual-source computed tomography scanner (SOMATOM Definition Flash, Siemens Healthineers, Forchheim, Germany).

The scanning parameters were: beam collimation 2 $\times$ 64 mm $\times$ 0.6 mm with a flying point on the z-axis, slice thickness 1.5 mm, tube voltage 110, current 300–450 mA and a reconstruction interval of 0.5 mm with electrocardiogram gating. Examination has been carried out using the prospective ECG-gated sequential scan technique. As already mentioned, a reduction in the dose of radiation in the middle of the heart cycle was introduced to limit the harmfulness of X-rays on tissues. Typically, the highest radiation dose is in the best systole and best diastole phase, which corresponds to 30 and 70% of the time elapsed between two successive R waves of the QRS signal on the electrocardiogram.

The methodology uses retrospective and anonymized patient imaging data acquired from routinely performed cardiac tomography. Based on this fact, in their letter of 18 July 2019, the Bioethics Committee of the Medical University of Silesia in Katowice deemed unnecessary ethics approval according to national regulation. The experiments were carried out according to the guidelines and regulations of the Silesian Center for Heart Diseases, Zabrze, Poland and the Declaration of Helsinki. Informed consent was obtained from all participants.

The patient was a 55-year-old male. A contrast bolus (Omnipaque 350) with a flow rate of 5 ml/s, a flow duration of 10.2 s, and a total dose of 50 ml was applied. In the case analyzed, the end systolic volume of the left ventricle was at the level of 28,85 ml, while during the end diastole it reached 137,49 ml. The calcium score for the investigated case was at the level of 5.2. At this level of the calcium score, the blooming effect is negligible^36^.

Two types of images were used. One series consisted of a high-resolution image set with 296 images of $512\times 512$ pixels, 0.4 mm pixel spacing (X and Y dimensions), and 0.6 mm slice thickness (Z dimension). These images were taken for the diastolic phase ($67\%$ phase of the cardiac cycle).

**Figure 1:**
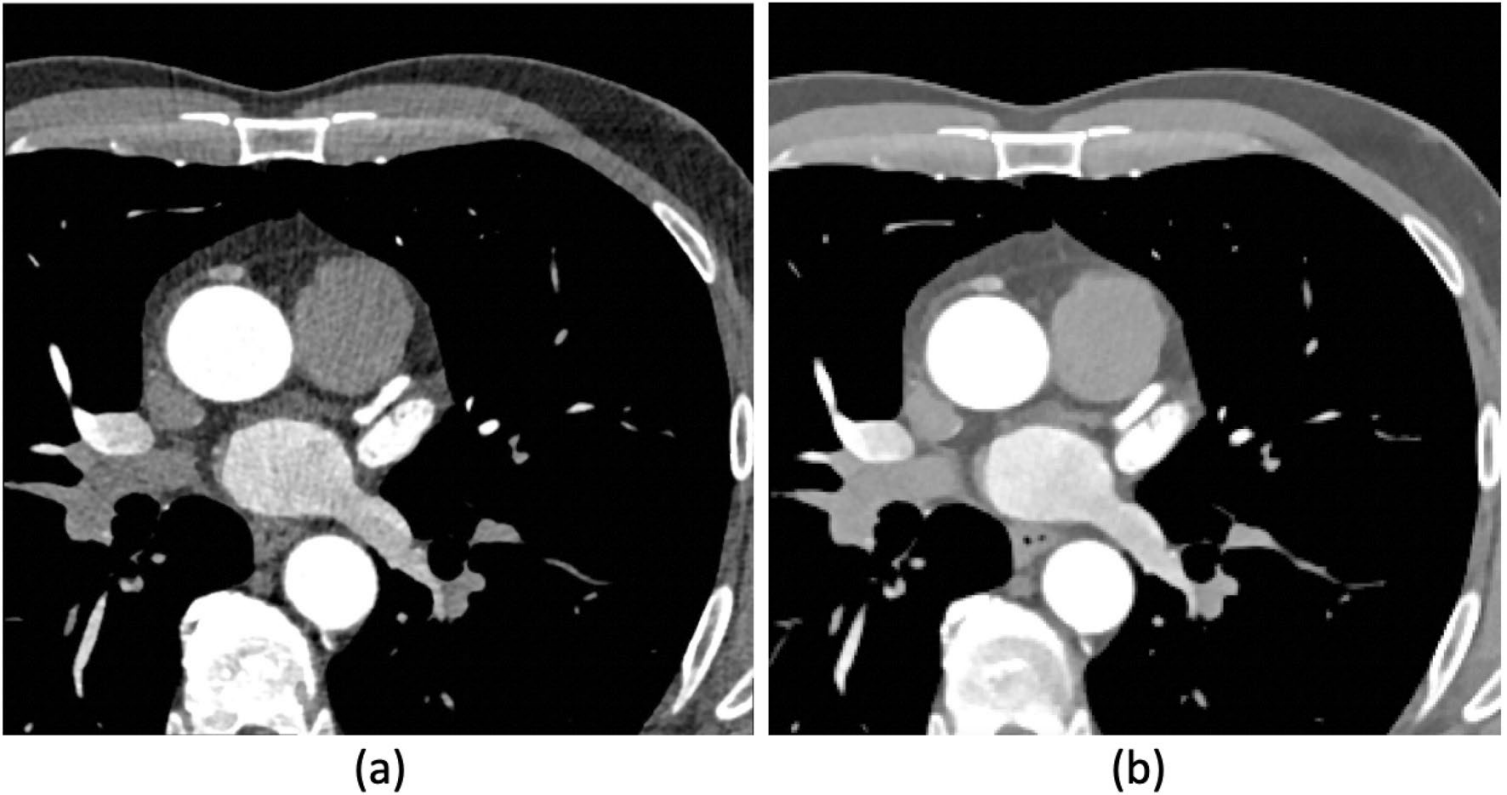
Sample slices from approximately the same location from high- and low-resolution Digital Imaging and Communications in Medicine file format (DICOM) data sets. (**a**) Slice from the high-resolution diastole dataset. (**b**) Slice from the low-resolution 70% phase of the heart cycle dataset.

The second series consisted of recording the dynamics of the heart cycle. Ten sets of ECG-gated images were acquired for the same field of view (FOV) as the high-resolution data, and the heart cycle was measured in a range of 10–100% phases of the heart cycle, with a step of 10%. For this series, image sets for each time step of 148 images were acquired with low-resolution $256\times 256$ pixels, 0.8125 mm pixel spacing (X and Y dimensions), and 1.5mm slice thickness (Z dimension). Sample slices of lower- and higher-resolution data sets are shown in Fig. 1. The image sets obtained near full cardiac diastole demonstrated very poor quality (*i.e.,* high noise presence). It is the result of the modulation of the applied intensity of the X-ray emission in different phases of the cardiac cycle.

**Figure 2:**
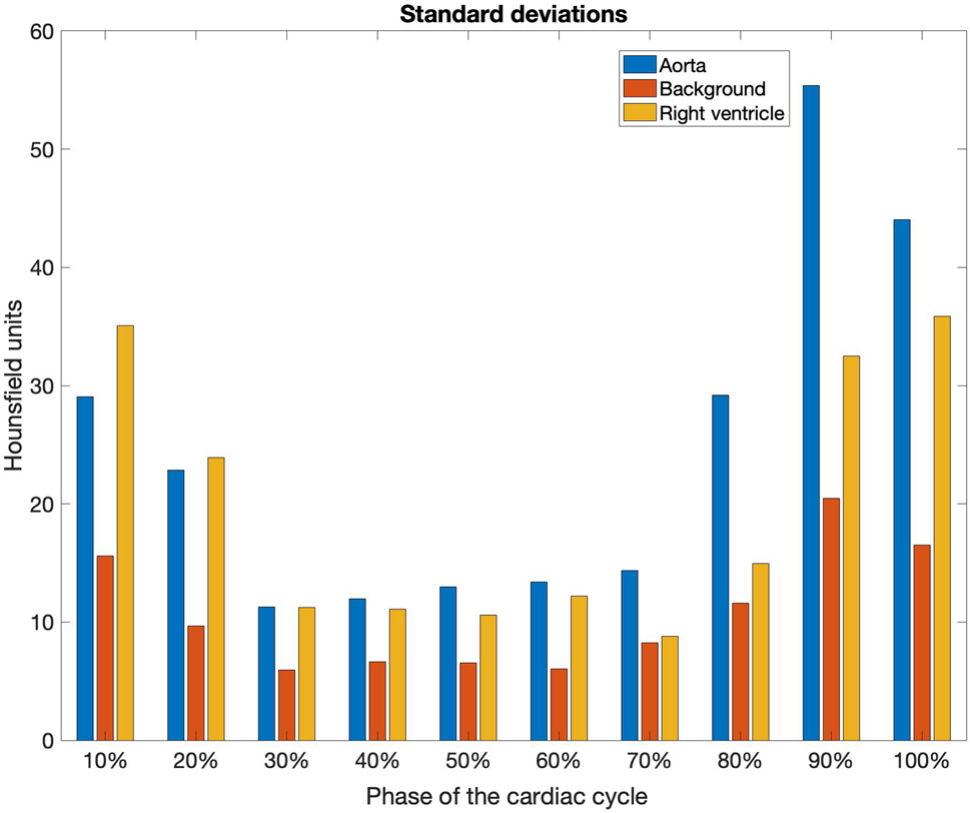
Distribution of standard deviation values from three distinct image areas: aorta (blue bars), background (orange bars) and right ventricle (yellow bars) for ten, successive phases of the cardiac cycle.

In order to measure the level of noise in the low-resolution images mean values and standard deviations were calculated for the three distinct areas: aorta, background and right ventricle. These areas have been selected due to their homogeneous nature in the CCTA modality. The mean values did not show variability in terms of the heart phase cycle (data not shown). The standard deviations showed variability depending on the phases of the cardiac cycle (see Fig. 2). The level of noise depends mainly on the imaging of the respective phase of the cardiac cycle and much less on the values of the Hounsfield unit describing successive regions. The lowest noise is present in the images for phases in the range 30–70%. The differences in image quality can be clearly seen in Fig. 6 where phases 60% and 90% are compared.

### The general workflow

**Figure 3:**
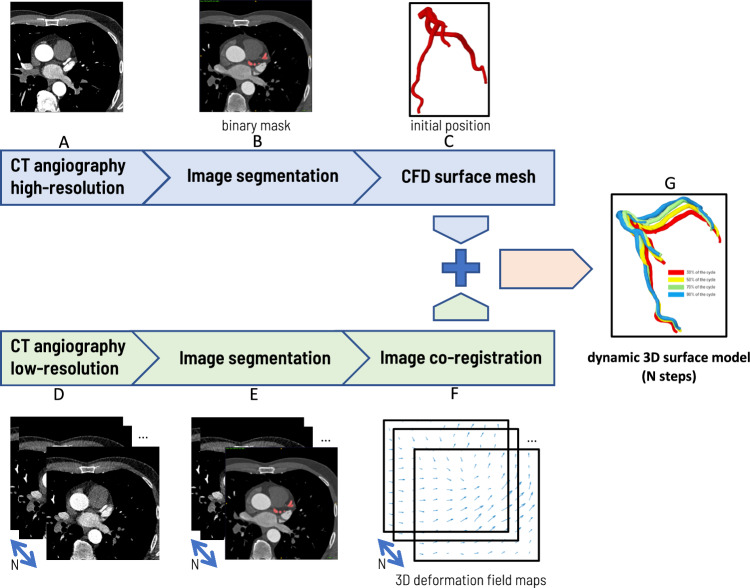
General workflow of the dynamic surface model of the blood vessels. Segmentation is performed for N = 10 phases of cardiac cycle.

The general workflow is presented in Fig. 3 and is described in detail in the following paragraphs.

The first step was image segmentation, which was based on the high-resolution image set (67% diastolic phase, $512\times 512$ pixels), resulting in a binary 3D mask (red areas in Fig. 3 B). This mask was used to produce the 3D (surface) model of the coronary arteries (specifically, the LAD artery). Then, a surface smoothing procedure was performed using Geomagic software^34^. The model, expressed as a set of points located on the surface of the vessel, has the form of a list of points (vertices of the mesh) along with their coordinates and the connectivity matrix. The latter defines how the vertices are connected to form the elements. This list of points (visualized in Fig. 3C) is then used to obtain a sequence of consecutive models resulting from the sequence of measurements taken in subsequent time steps (seen in Fig. 3G). For this purpose, all ten low-resolution image data sets were segmented and used (Step E in Fig. 3). Based on these images, 3D transformations were calculated using image co-registration algorithms (step F in Fig. 3). These transformations were used sequentially to modify the coordinates of the 3D surface mesh. This procedure produced a sequence of the transformed initial set of points (and their X, Y, and Z coordinates) to reflect the dynamics of the LAD structure.

### Image segmentation

Segmentation of the coronary arteries by CT angiography is a challenging task. Among the methods described in the literature, three distinct classes can be distinguished^37^: region-growing methods, active contours (including snakes and level-set-based approaches), and centerline-based methods. Recently, deep learning methods have been used, including convolutional neural networks (CNN) for coronary vessel segmentation^38^.

In the present work, the focus was not on automating the segmentation process. This refers especially to low-resolution data of successive phases of the cardiac cycle, where segmentation without operator involvement would be very inefficient.

Segmentation was performed using the ITK-SNAP software^33^ by means of an active contour method with a threshold-based pre-segmentation mode. This 3D active contour segmentation method captures the evolution of the closed surface *C*(*u*, *v*, *t*) parameterized by variables *u*, *v*, and the time variable *t*. The contour dynamics is described by a partial differential equation:

2 $$\begin{aligned} \frac{\partial }{\partial t} C(u,v,t) = F \, \vec {n}, \end{aligned}$$

where $\vec {n}$ is the unit normal to the contour, and *F* represents the sum of internal and external forces that act on the contour in the normal direction. In our case, the internal force is associated with the mean curvature of *C* and external with the magnitude of the gradient of the intensity of the image. The force acting on the contour has the form

3 $$\begin{aligned} F = \alpha g_I + \beta \, \kappa , \end{aligned}$$

where $g_I$ is the speed function derived from the magnitude of the gradient of the input image *I*, $\kappa$ is the mean curvature of the contour, and $\alpha$ and $\beta$ are the weighting coefficients of particular forces. The speed function may be defined as follows:

4 $$\begin{aligned}{} & {} g_I(\xi ) = \frac{1}{1 + (NGM_I(\xi )/v)^\lambda } , \end{aligned}$$

5 $$\begin{aligned}{} & {} NGM_I(\xi ) = \frac{\Vert \nabla (G(\xi )_{\sigma } * I(\xi )) \Vert }{max_I\Vert \nabla (G(\xi )_{\sigma } * I(\xi )) \Vert } , \end{aligned}$$

where $NGM_I$ is the normalized gradient magnitude of the image $I(\xi )$; $(G_{\sigma } * I)(\xi )$ denotes the convolution of $I(\xi )$ with the isotropic Gaussian kernel ($G(\xi )_{\sigma }$) with standard deviation $\sigma$, and *v* and $\lambda$ are parameters that determine the shape of the monotonic mapping between the normalized gradient magnitude and the speed function. The role of the speed function is to take values close to 0 at the edges of intensity in the input image, and values close to 1 in regions where intensity is nearly constant.

This method requires the user to mark seed points, and it is often necessary to manually correct the segmentation results, especially in the area where the LCA is close to the left atrial appendage. The threshold for pre-segmentation was set to 166 Hounsfield units. The parameters chosen heuristically for the evolution of the active contour were the following: the region competition force $\alpha =0.95$ and the smoothing force $\beta =0.2$. The number of iterations of contour evolution varied according to the size of the data sets and the number of seed points. The remaining parameters related to the speed function (4), namely $\sigma$, *v*, and $\lambda$, are internal ITK-SNAP parameters, which cannot be manually set by the user.

### Image co-registration

Co-registration of the CT image data sets was performed to obtain the transformations necessary to modify the 3D surface model points of the coronary arteries. To obtain the appropriate transformation maps, all temporally contiguous measurements have been co-registered using the ANTs software package (version 2.3.5)^35^. For transformations, three stages were used:

- Rigid
- Affine
- Deformable B-spline SyN

These transformations were implemented using the built-in script antsRegistrationSyN.sh with its predefined parameters. The last deformation step used the symmetric diffeomorphic algorithm (so-called Symmetric Normalization SyN) proposed by Avants et al.^39,40^. This multistep approach produced two important pieces of information: the affine transform matrix (in the format of a .mat file) and the deformation field matrix, resulting from the non-rigid registration step. The deformation field was stored as a 4D matrix consisting of 3D deformations in all three dimensions. Both sets of information were needed to transform the coordinates of the mesh points from the initial configuration to the current configuration. Next, the antsApplyTransformsToPoints script with its predefined control parameters, from the ANTs package, was used to modify the coordinates of the points. As a result of the image co-registration step, multiple sets of point coordinates were produced and stored in .csv file format. The number of sets of outputs depended on the number of steps produced by CT within a cardiac cycle and taken for analysis.

An important aspect of performing the correct co-registration and transformation of points to the new space is to work in the same coordinate system. Particular attention should be paid to the way the coordinate system is read by the software used because it can be a source of difficulties in the implementation of the method. The use of different environments such as Matlab, ITK library, Python, or ANTs in a single framework allows for a great deal of freedom in data manipulation, but also results in an often unintentional change of the coordinates.

For example, it must be taken into account that the ITK coordinate system differs from the one defined by Nifti, where the *X* and *Y* coordinates are reversed, while the *Z*-axis remains the same. ANTs software uses ITK libraries, so it maintains the same coordinates, whereas when reading the .nii files in Matlab or Python, the coordinates need to be transformed.

In the present work, we used the coordinate system read by ITK, which was our preferred reference system. This fact is crucial for the transformation of points in the surface model (also stored in the correct coordinate system) when the deformation map is also defined in this system.

### Quality assessment

To check the quality of segmentation, as well as the entire process of creating new 3D surface objects representing coronary arteries, the quality measures Jaccard index (JAC)^41^ and the Dice coefficient (DICE)^42^ were used. These measures are defined by equations (6) and (7), respectively. Such measures quantify the accuracy of the coverage of the reference area by the segmentation area. Both measures reach a minimum of 0 for completely disjoint sets and a maximum of 1 for perfect coverage.

Jaccard index is expressed by the following equation:

6 $$\begin{aligned} JAC = \frac{|A \cap B |}{|A \cup B|}, \end{aligned}$$

where $| \cdot |$ denotes the cardinalities of sets *A* and *B*, $\cap$ denotes the intersection of two sets, and $\cup$ denotes the union of them.

The DICE is expressed by the following equation:

7 $$\begin{aligned} DICE = \frac{2|A \cap B |}{|A|+|B|}, \end{aligned}$$

where $| \cdot |$ denotes the cardinalities of a set *A* and *B*, and $\cap$ denotes the intersection of two sets.

Both measures are related to each other and their dependence can be expressed by the following formula: $JAC = \frac{DICE}{2-DICE}$.

## Results

### Segmentation results

**Figure 4:**
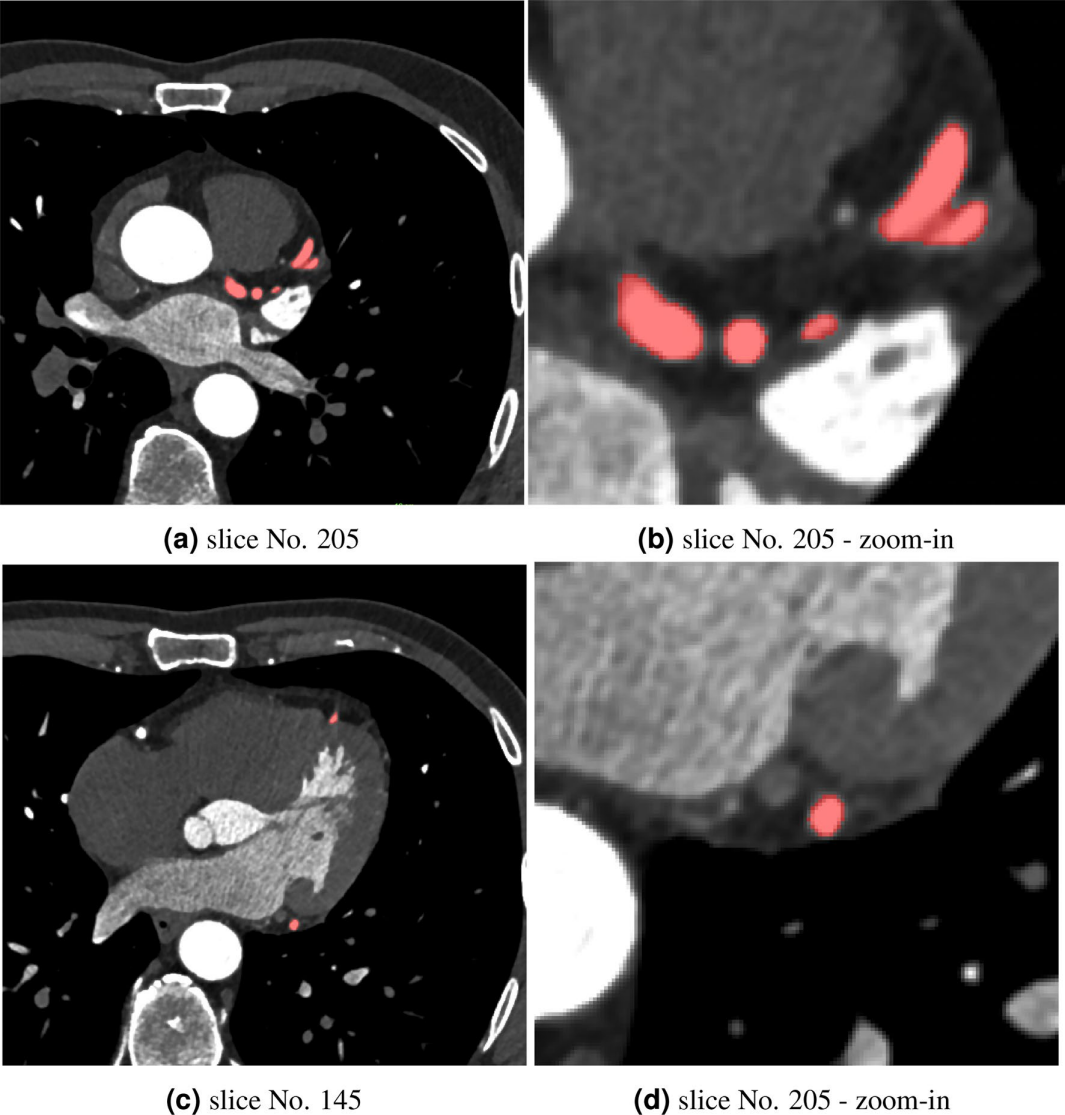
Segmented arteries overlaid on the CT images. Image (**a**) shows data from slice No. 205. Image (**b**) presents a zoom-in on the arteries of interest. Image (**c**) shows data from slice No. 145, and image (**d**) presents a zoom-in on the arteries of interest.

The results of the segmentation of high-resolution images are shown in Fig. 4. Segmented vessels of the left coronary arteries are colored red in the two selected cross sections.

**Figure 5:**
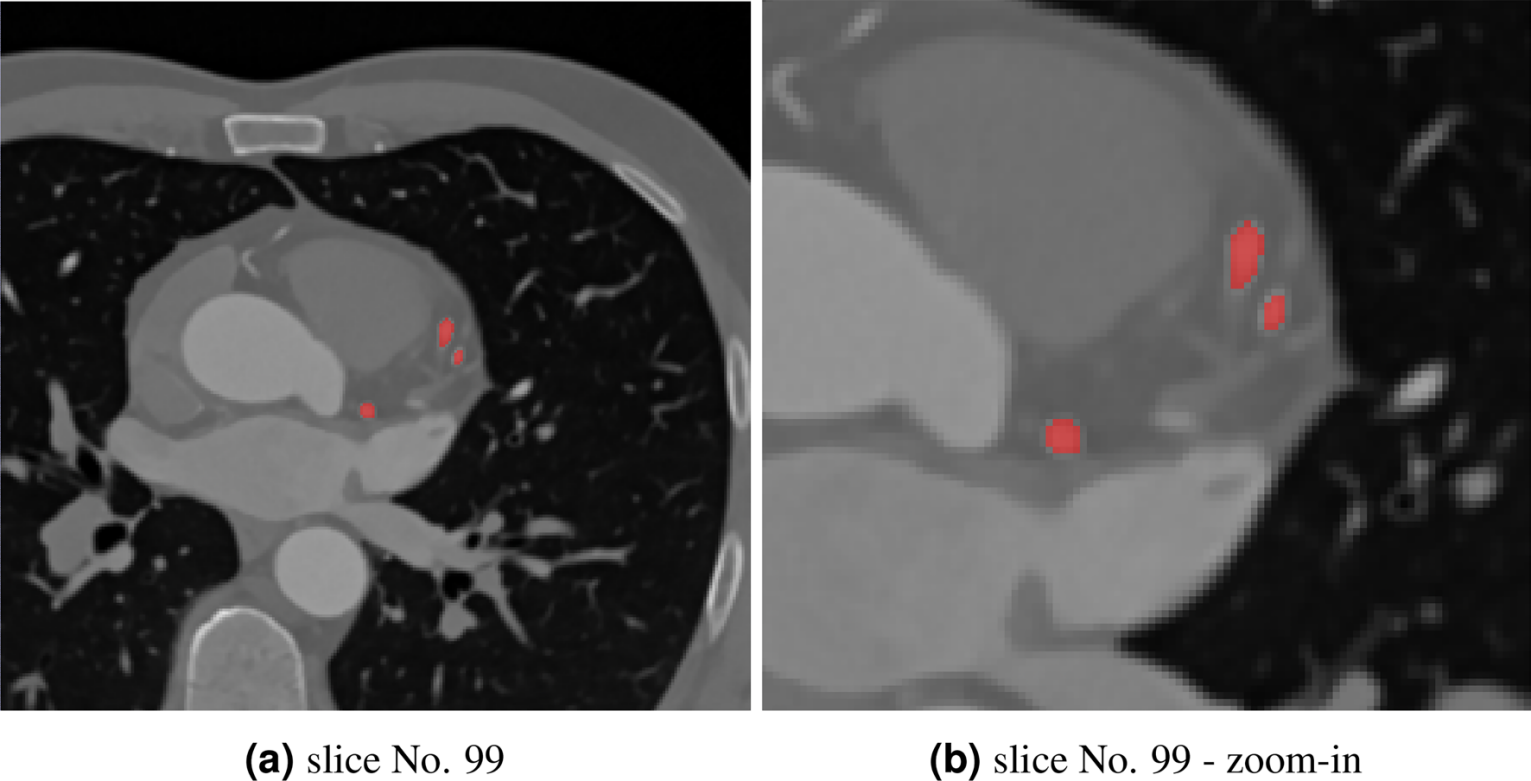
Segmented arteries overlaid on the low-resolution CT images (70% phase of the cardiac cycle). Image (**a**) shows data from slice No. 99. Image (**b**) presents a zoom-in on the arteries of interest.

The sample results for the segmentation of lower-resolution images are presented in Fig. 5. Here, the segmented vessels of the left coronary artery are also shown in red.

**Figure 6:**
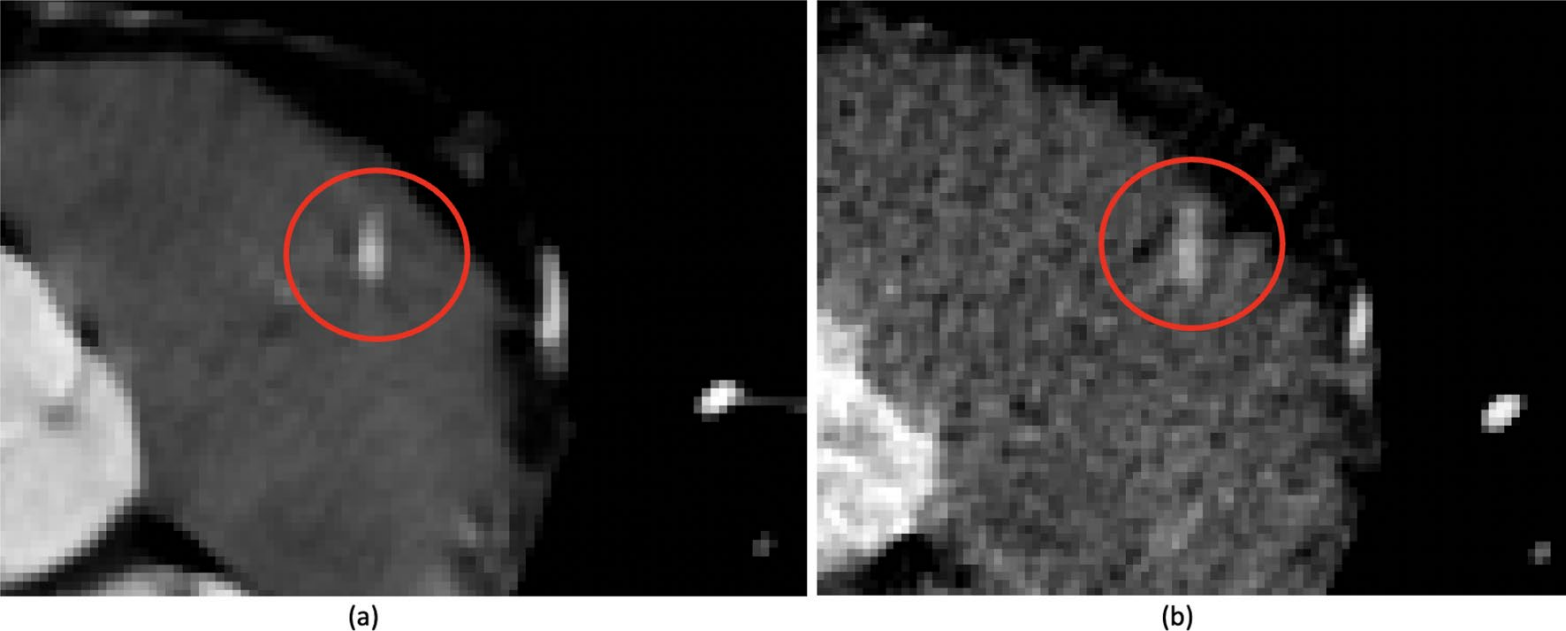
Comparison of two images with the LAD marked by a red circle. (**a**) Image at the 60% phase of the cardiac cycle. (**b**) Image at the 90% phase of the cardiac cycle.

Segmentation of the data sets for each phase of the heart cycle (in total 10 phases) proved to be more demanding. This was because images closer to the full diastolic phase were very noisy. In these cases, manual correction of the segmentation results was often required in places where the boundary between the artery and the surrounding tissue was barely distinguishable (see Fig. 6).

### Creation of a dynamic 3D model

The first step of the procedure was the segmentation of low-resolution images corresponding to all ten phases (i.e. 10, 20,..., 100%) of the cardiac cycle, schematic representation is shown in Fig. 3E. The next step was to determine the geometry transformations of the subsequent adjacent in-time images (Fig. 3F). The transformation consisted of defining the affine transformation matrix and defining the vector field the diffeomorphism. These two operations, which convert the segmented object to the adjacent one, were performed in the ANTs package^39,40^.

In the next step, the high-resolution image segmentation corresponding to the 67% phase of the cardiac cycle was performed (Fig. 3B). The result of the segmentation was then smoothed using GeoMesh^34^, producing a 3D domain. Using the standard ANSYS-Fluent^43^ CFD mesher, a volumetric CFD numerical grid was generated in this domain. A set of user-defined functions was then invoked to select the nodes on the boundary of the 3D object to retrieve the topology of the mesh (connectivity matrix). These nodes were then transformed using predefined affine transformation matrices and deformation vector fields corresponding to the 10, 20,..., 100% phases of the cycle (Fig. 3G). Because the CFD nodes were present in a high-resolution image, their transformation to low-resolution objects required spatial interpolation. This operation was carried out by invoking the procedure antsApplyTransformToPoints included in the ANTS package.

The CFD surface mesh (nodes with identical topology in all objects) was obtained upon completion of the steps described above. Generation of CFD meshes between known grids for the 10, 20,..., 100% phases of the cardiac cycle was performed by time interpolation. The identical mesh topology in all objects allowed us to use the dynamic mesh option (mesh morphing) of the CFD solver.

Some explanation is required for the use of a high-resolution image in the described procedure. This image, by its very nature, provides more accurate information about the shape of the arteries under examination. This allows for a better representation of arterial shape at times of the cardiac cycle when only low-resolution images are known. However, if a high-resolution image is not available, the procedure can work only on low-resolution images.

**Figure 7:**
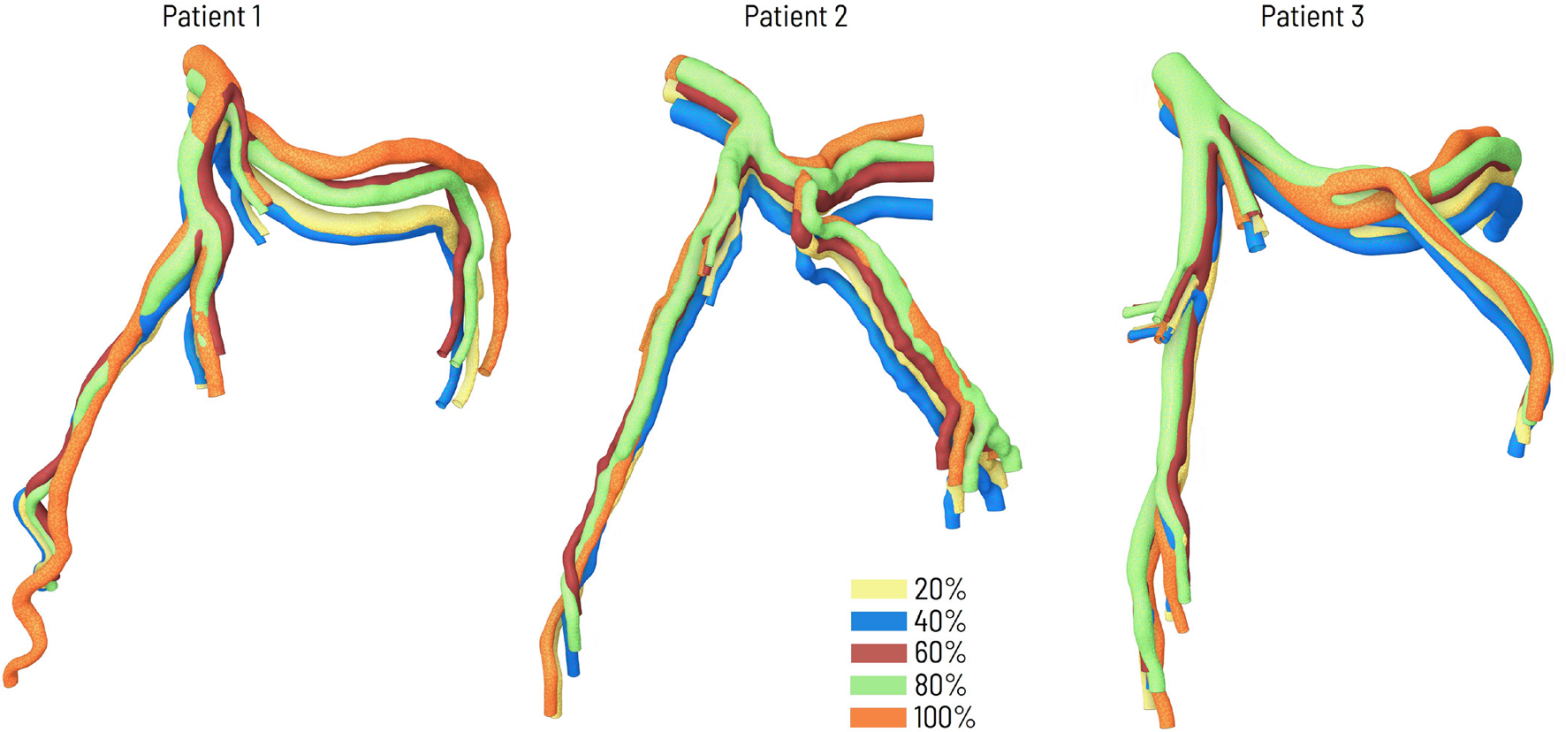
Geometries of three patients presented as ANTS results for selected time steps.

Although the paper is intended as a case study for Patient 1, the methodology developed was applied to the angio-CT images of three patients, resulting in similar precision. Figure 7 shows the temporal variation of the geometry of the arteries at selected time steps of the cardiac cycle for these three patients.

In the supplementary online materials (Supplementary Data 1), a video file (https://zenodo.org/doi/10.5281/zenodo.10203115, minimum play resolution is HD to see the mesh) can be found showing the movement of the LCA throughout the heart cycle. This animation was performed using four positions of blood vessels, and all stages between them were the results of linear interpolation in time. Using this method, it is possible to generate an infinite number of intermediate positions of blood vessels. In this case, the proposed linear interpolation between 3D shapes in time is a simplification, and other methods such as higher-order polynomial interpolation could also be applied. In addition, files for the LCA surface model are also available in .stl format. These and other raw data files are available as supplementary material (Supplementary Data 1).

### Segmentation validation

#### Validation of high-resolution CT angiography image segmentation

An experienced cardiologist (co-author MK) validated the results of coronary segmentation performed on the CT data. The expert manually found the vessel contours for several planes created from the multiplanar reconstruction (MPR) projections. Using Syngo.VIA software (Siemens), the expert marked and saved arterial contours in a plane perpendicular to the centerline of the vessel. All necessary information on the transformation of contour point coordinates from 2D planar projections to their 3D locations in the patient coordinate system was saved in the DICOM (Digital Imaging and Communications in Medicine) headers. For the selected patient, 18 contours were produced in the volume of the artery of interest.

To compare the cross-sections from the MPR projection with the results of semi-automatic segmentation, an inverse transformation of the vessel’s edge points from the 3D space to the appropriate 2D projection was necessary. Relevant cross-sections, corresponding to those recorded by the expert, were found and compared with the semi-automatic contours produced in the previous step. The edge points were calculated from the 3D surface model (isosurface calculated in the Matlab environment from segmented arteries) being cut in the same plane as the contours made by the expert. For this purpose, we applied the routine by J. Tuszynski^44^, which is based on the two triangle intersection method by T. Möller^45^.

**Figure 8:**
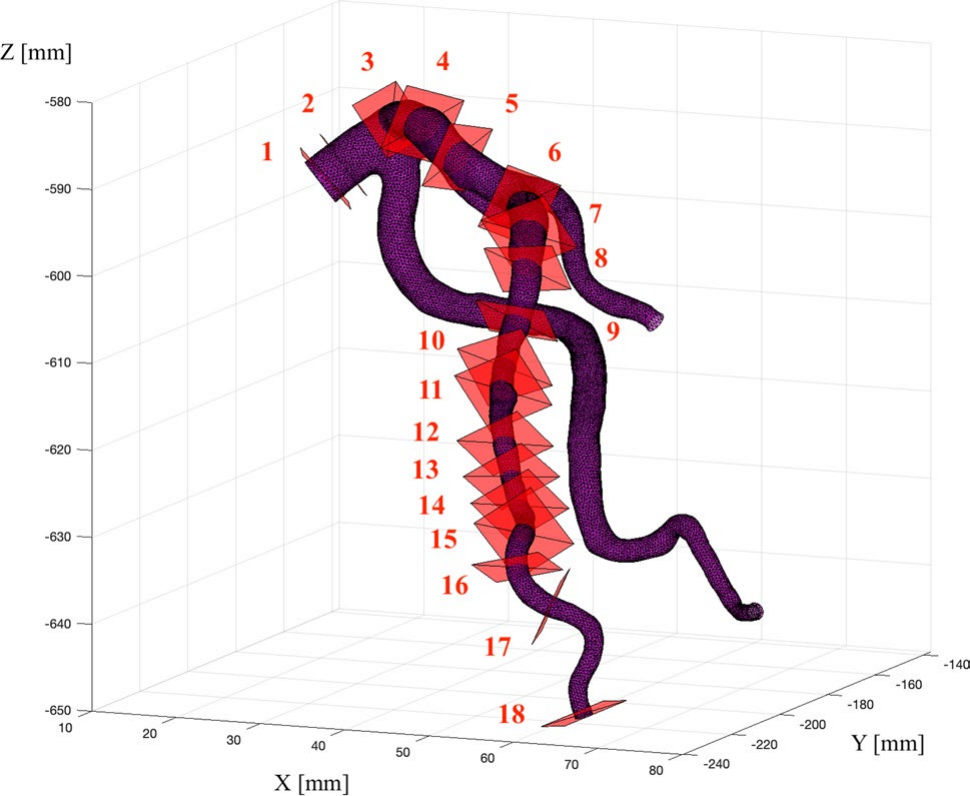
Surface model of the LCA for heart diastole with 18 visible rectangles representing the cutting planes. The red numbers indicate the numbering of the respective cutting planes.

Visualization of our LCA surface model with 18 cutting planes represented by rectangles is presented in Fig. 8. Validation was limited to the branch of the LCA shown in Fig. 8, because the MB was present only in the LAD for this particular patient.

For the 18 cutting planes, the quality measures JAC and DICE were calculated, and their values for successive planes are presented in Table 1. The results deteriorated with the decrease in the diameter of the vessel.

**Table 1:** Comparison of quality measures of the surface model for successive artery-cutting planes. The number of cut plane positions increases with the distance along the vessel, starting from its widest diameter.

Position	JAC	DICE	Position	JAC	DICE
1	0.92	0.96	10	0.74	0.85
2	0.90	0.95	11	0.72	0.84
3	0.70	0.82	12	0.55	0.71
4	0.81	0.89	13	0.60	0.72
5	0.76	0.86	14	0.66	0.79
6	0.80	0.89	15	0.78	0.88
7	0.84	0.92	16	0.94	0.97
8	0.78	0.88	17	0.76	0.86
9	0.64	0.78	18	0.85	0.92
Mean ± SD		JAC: 0.76 ± 0.11		DICE: 0.86 ± 0.07	

However, it should be kept in mind that the calculated measures were relative (dimensionless) and did not take into account physical conditions. Both the segmentation error and the expert’s contouring error increase as the diameter of the artery decreases, which have a significant impact on the error of the calculated similarity measure. For thin arteries with a diameter of approximately 1.6 mm, a change of one pixel (where a pixel has a physical dimension of $0.40625 \times 0.40625$ mm) can generate about a 25% diameter error.

#### Validation of lower-resolution image segmentation and 3D coronary model creation results

Similarly, a validation was performed for low-resolution images. However, we had a limited number of cross sections with contours marked by the expert for these images. Validation was carried out in four phases of the cardiac cycle (30, 50, 70 and 90%) for three MPR projections in each of the phase.

**Figure 9:**
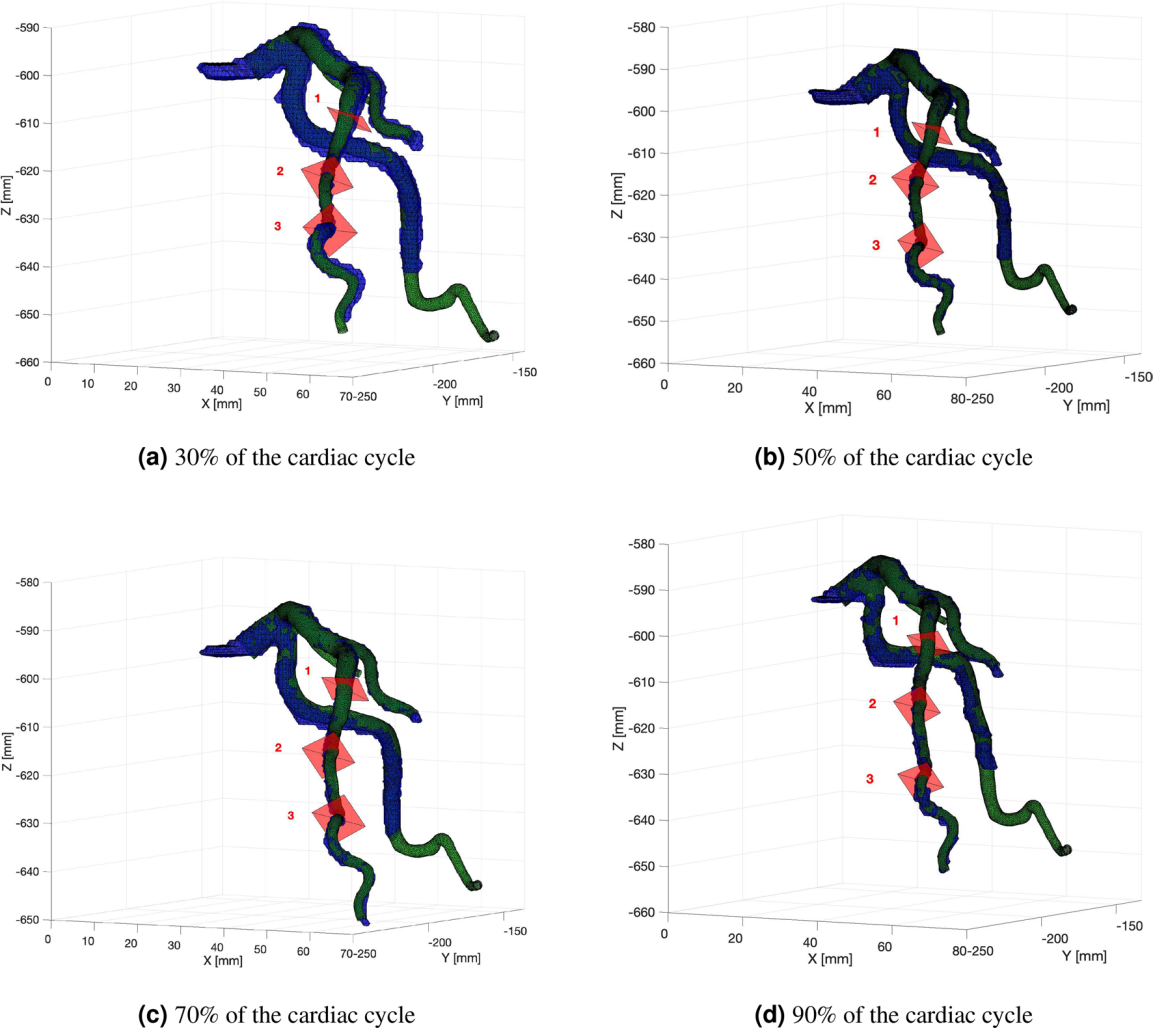
Surface models for different phases of the cardiac cycle showing three cutting planes. Figures show the left coronary arteries during (**a**) the 30%, (**b**) 50%, (**c**) 70%, and (**d**) 90% cardiac cycle phases. Surfaces in blue are generated from segmented images, while the green surfaces are generated from data transformed in time and space.

**Figure 10:**
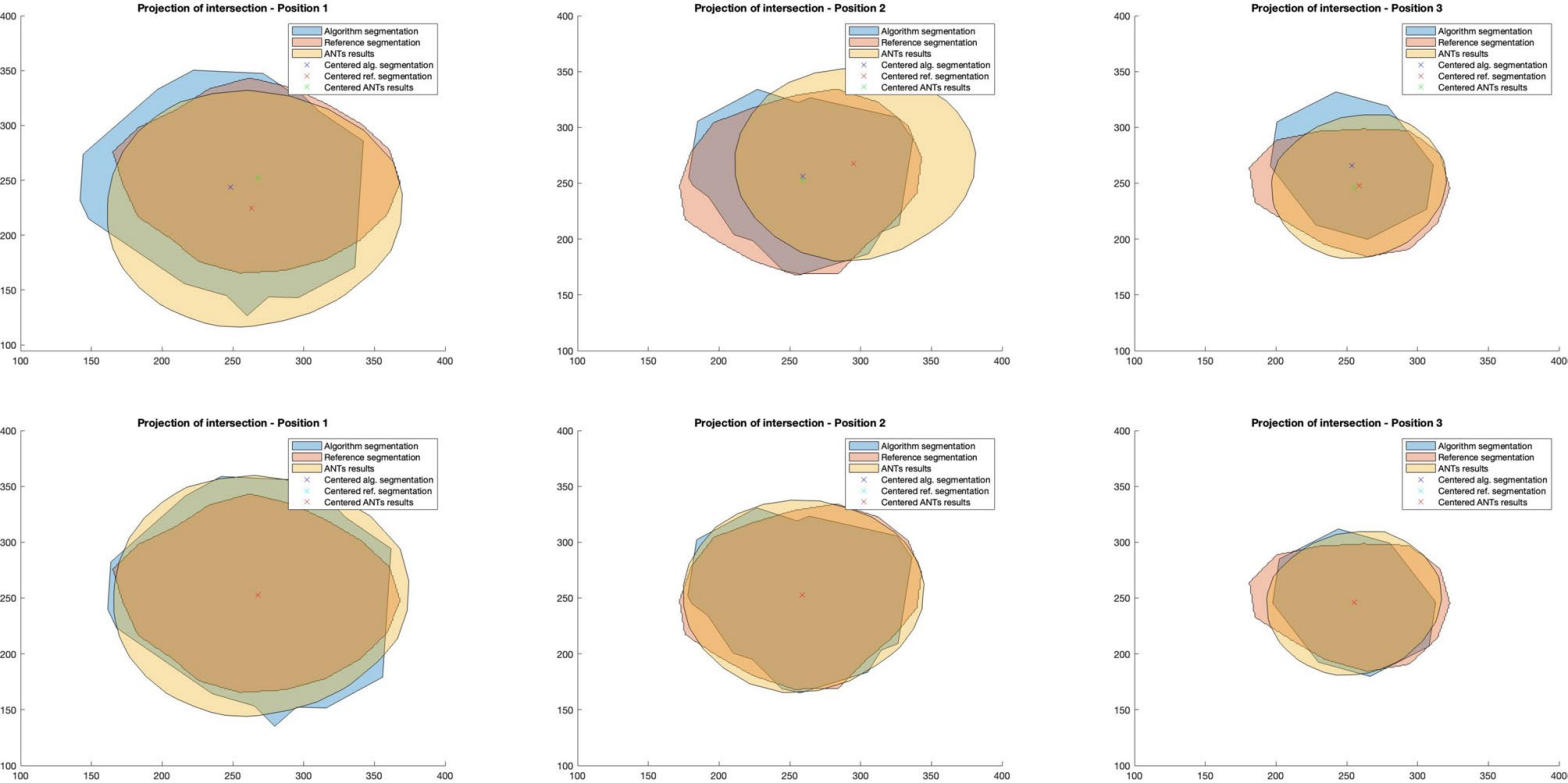
Comparison of vessel edge shapes for the 50% cardiac cycle phase shown in local coordinates of the appropriate section planes. Different colors present: algorithm segmentation (blue), reference segmentation (rose), deformed with ANTs segmentation (yelow). The upper subplots present vessel edge shapes in original positions, and the lower subplots present vessel edge shapes after centering. In rows are presented three different positions of vessel edge shapes.

The visualization of our LCA surface model is shown in Fig. 9 with three cutting planes represented by rectangles. In addition, a visual comparison of the surface areas of the vessel at the cutting sites is presented in Fig. 10.

For the four low-resolution data sets for which three MPR data with marked contours were produced, the quality measures JAC and DICE were calculated, and their values for successive planes are presented in Table 2. This table contains both the quality measures for segmentation and the results of the ANTs algorithm. All measures were then recalculated for the case of aligned centers of gravity of the compared contours. The aim was to check whether the lower quality measures were due to inaccuracy in shape reproduction or imprecision of its location along the vessel. From the values of the measures shown in Table 2 and images presented in Fig. 10, it is clear that the diameters of the vessels are preserved, while there are differences in the accurate representation of the position of the arteries (see the columns Segmentation and ANT versus Segmentation and ANT centered in Table 2). These errors are particularly evident for the most distant parts of arteries (it can be seen at position 2 or 3 for each cardiac phase in Table 2).

**Table 2:** Comparison of quality measures for different phases of the cardiac cycle. In the subsequent rows, different phase stages (30, 50, 70, and 90% are presented for the case examined). Two values of quality measures are shown in columns JAC and DICE for segmentation, registration result with ANTs software, centered segmentations, and centered registration results.

Phase	Position	Segmentation		ANTs		Segm. centered		ANTs centered	
		JAC	DICE	JAC	DICE	JAC	DICE	JAC	DICE
30%	1	0.68	0.81	0.50	0.67	0.72	0.84	0.82	0.90
30%	2	0.60	0.75	0.72	0.83	0.60	0.75	0.72	0.83
30%	3	0.65	0.79	0.63	0.77	0.68	0.81	0.83	0.91
50%	1	0.67	0.81	0.67	0.80	0.78	0.88	0.72	0.84
50%	2	0.83	0.91	0.54	0.70	0.83	0.91	0.89	0.94
50%	3	0.59	0.74	0.81	0.90	0.77	0.87	0.81	0.89
70%	1	0.74	0.85	0.74	0.85	0.82	0.90	0.75	0.86
70%	2	0.80	0.89	0.43	0.60	0.86	0.92	0.70	0.83
70%	3	0.63	0.77	0.78	0.88	0.84	0.91	0.84	0.91
90%	1	0.72	0.84	0.69	0.82	0.77	0.87	0.69	0.82
90%	2	0.79	0.88	0.51	0.68	0.85	0.92	0.63	0.77
90%	3	0.52	0.69	0.43	0.61	0.53	0.70	0.53	0.69
Mean		0.69	0.81	0.62	0.76	0.75	0.86	0.74	0.85

### Preliminary CFD results

**Figure 11:**
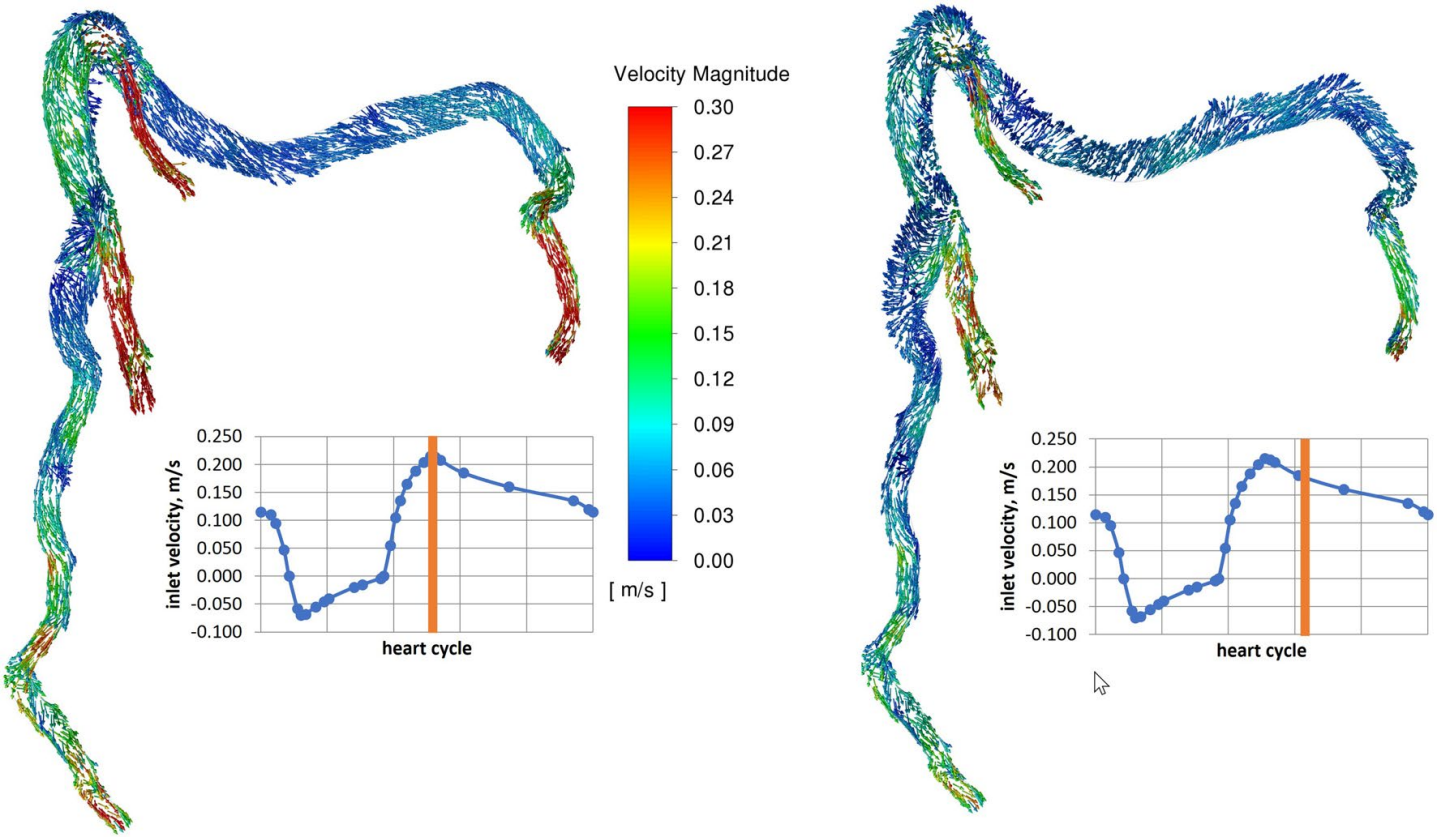
Velocity vectors in m/s for two time steps representing 50 and 68% of the heart cycle.

CFD analysis was performed to assess the applicability of the methodology presented in the previous sections. The dynamic mesh with constant topology of the external surfaces allowed us to perform numerical simulations on the basis of the ANTs results. Using the generated mesh, a CFD problem was solved. The resulting velocity field for the first patient is depicted in Fig. 11 at 50% and 68% of the cardiac cycle.

The results of the CFD simulation (velocity fields) of the selected patient at three moments of the cardiac cycle are included in the article. Calculations were made using the ANSYS Fluent package on the Dell Precision 3630 Tower (Intel(R) Core(TM) i7-9700 CPU @ 3.00 GHz). In the presented case, the grid consisted of 326575 volumetric elements (the average orthogonal quality (OQ) was 0.74 and the minimum OQ was 0.04). The number of nodes defined by the ANTS algorithm reached an approx. 19k elements, and the positions of those elements were defined by the set of UDFs in each time step. The rest of the nodes in the numerical mesh were adapted by the internal software procedure based on smoothing and remeshing methods available in Ansys Fluent.

Moreover, for the presented case, the fine mesh was also investigated reaching 1087868 volumetric elements. The minimum OQ for this mesh reached 0.1 while its average value was at the level of 0.78. This fine mesh produced similar dynamic geometry, and the presented procedure was repeatable for this grid. The objective of the simulations was to check the possibility of CFD analysis in vessels whose shape changes in time. The dynamic simulation of the shapes of the coronary arteries aims to improve the accuracy of blood flow estimation. However, in most cases, it is stenosis that controls hemodynamics. The presented results were obtained applying simplified, constant pressure outlet boundary conditions of 75 mmHg (diastole level).

The detailed analysis of the obtained field of shear stress, its oscillations, residence time, and its comparison with the observed calcification regions, as well as the applied boundary condition, will be the topic of a subsequent paper.

Although the paper is intended as a case study, the methodology developed was applied to the angio-CT images of three patients, resulting in similar precision.

## Discussion

The results presented here demonstrate our proposed workflow to obtain the dynamics of a 3D vessel model. The steps include a segmentation step (for both high- and low-resolution images) of the CT images, image co-registration taking into account several steps in the cardiac cycle, creation of a surface model of the vessel, and transformation of the points according to previously obtained deformation maps.

The first concern is the accuracy of the segmentation algorithms, especially when using low-resolution images with a high values of standard deviations. The quality of the data, as shown by the degree of image noise, varied at different times of the cardiac cycle, as shown in Fig. 2. The best of the image sets are those in phases ranging from 30 to 70%.

This condition and the small volume of the segmented object, that is, the vessel, required numerous manual corrections of the result of the automatic segmentation. In particular, the plaque was visible at a location where the left circumflex artery (LCX) was close to the left atrial appendage (LAA). The validation of the segmentation result using manually produced contours by the expert cardiologist showed that the best results were for the widest vessels (which was expected) where there was a MB (see Table 1).

Another important processing step was the registration of segmented vessels. Our first attempts at co-registering raw CT images did not yield satisfactory results. Too much information and a small volume of vessels resulted in a very poor co-registration result. This could be fixed by using the result of vessel segmentation instead of the raw data.

Vascular segmentation at each step of the cardiac cycle produced different lengths and volumes of the segmented vessel. This effect was due to differences in image quality in successive imaged steps of the cardiac cycle.

**Figure 12:**
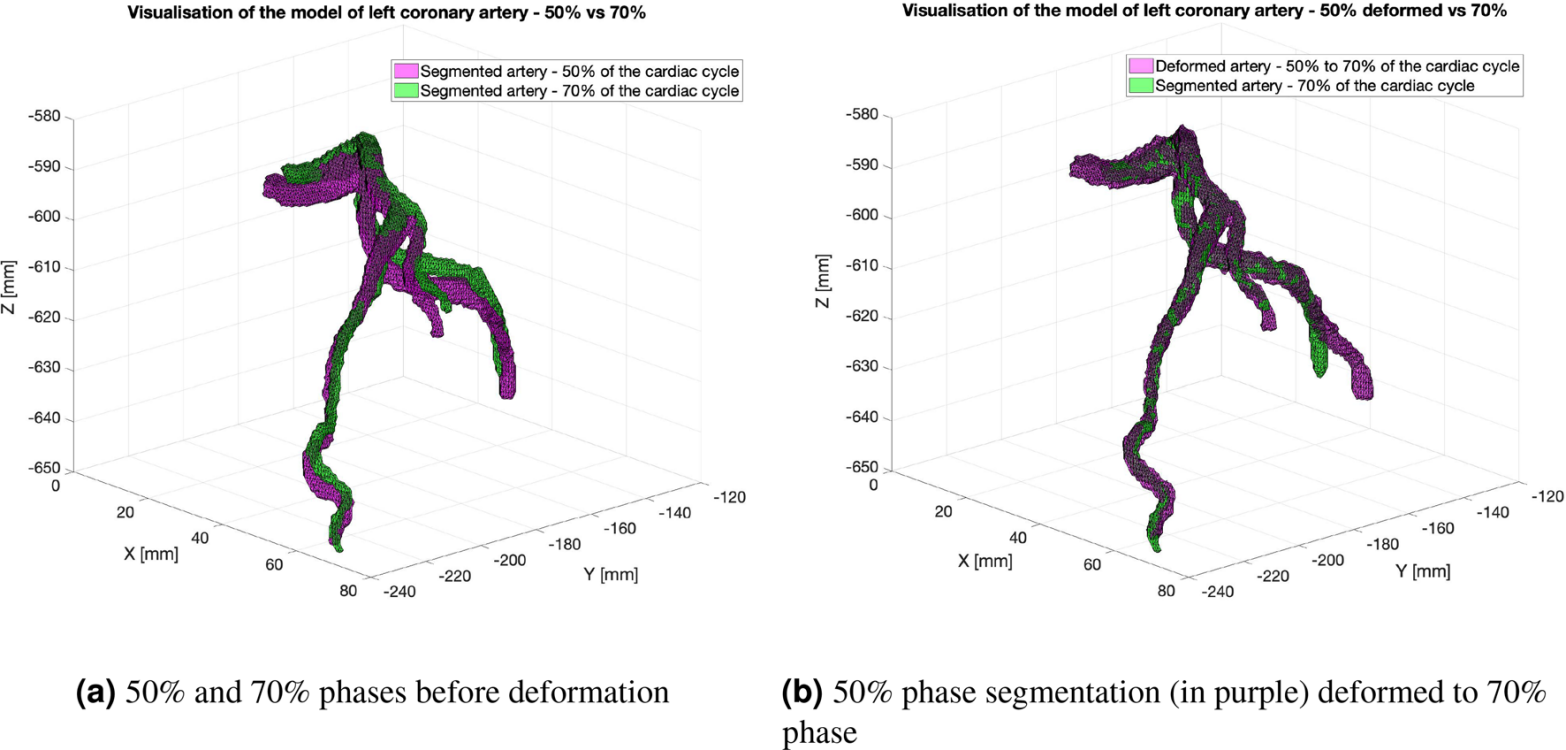
Visualization of the left heart artery model—comparison of the segmentation and ANTs results for the 50% and 70% phases of the cardiac cycle. Left plot: artery in both phases before deformation, right plot: 50% phase segmentation (in purple) deformed to 70% phase segmentation (in green).

Due to the aforementioned small vessel volume and small cross-sectional diameter, as well as differences in the volume of the segmented vessels at each step of the cardiac cycle, the results of the co-registration were also not perfectly matched, especially at the ends of the segmented volumes. This effect can be observed in Fig. 12, where the left side shows two volumes, namely at the 50 and 70% phases of the cardiac cycle. The right subfigure presents the effect of the co-registering stage from the step 50% phase to the step of 70% phase. However, the obtained deformations were sufficient to effectively transform the grid points of the surface model derived from the segmentation of the vessels in the high-resolution CT image.

When analyzing the quality of the overlay carried out in the ANTs software, the largest errors were concentrated at the ends of the segmented vessels. This was due to the small diameter of the vessel cross-sections at the ends of the imaged volume and the different volumes and lengths of the segmented vessels, particularly at their ends. To minimize this error, the images corresponding to subsequent phases of the cardiac cycle were clipped. The idea was to start at LCA ostium and end at a characteristic feature of the vessel. It should be stressed that the lengths of the segments at a given time interval vary, which results from the deformation of the myocardium within the cardiac cycle.

It is also important to note that the results of vessel segmentation were used for the co-registration, instead of the raw data. The use of the full image volume data from the CT study resulted in an inaccurate co-registration outcome and was not suitable for use in this context.

Again, it is worth highlighting the importance of using a single identical coordinate system for all sets used in the processing. This is especially important if the processing is performed using different software.

The hemodynamics is, to a great extent, controlled by the diameter (stenosis) of the vessels and not their curvature. The developed method traces changes in the diameter of the vessels, and thus it can reproduce the shape of the artery within the MB.

## Conclusions

In this paper, we propose a method for generating numerical meshes for a CFD solver to be used in simulations of blood flow in deformable vessels, for which 4D medical images are available. Although the paper is intended as a case study, the developed methodology was applied to the angio-CT images of three patients, resulting in similar precision.

The novelty of the proposed methodology is as follows:

- Development of an original methodology for the generation of a sequence of time-dependent 3D shapes of vessels. The procedure is coupled with a CFD mesher and produces a surface mesh of intact topology, suitable as a mesh morphing utility. The input of this procedure is a sequence of raw 3D images taken at successive times.
- The procedure accepts the presence of images of different resolutions and quality in the input sequence.
- Both the intermediate shapes of the vessels, for which no images are available, and the corresponding numerical mesh can be readily obtained by interpolation.

Important practical hints resulting from the research:

- The first set of nodal points used by ANTs that will be projected on subsequent vessel shapes should be generated on the smoothed surface of the segmented vessel.
- The software used for segmentation and ANTs should use the same coordinate system.
- The procedure better reproduces the shape of the cross-section of the vessel than the location of its center.
- The developed technique can be applied to generate numerical meshes in the arteries, heart, and other organs whose shape changes over time.

### Supplementary Information

Supplementary Information.

## Data Availability

A video file (minimum play resolution is HD to see the mesh) showing the movement of the LCA throughout the heart cycle and .STL files for 10–100% (increment of 10%) of the heart cycle phases are available to download from the open repository (10.5281/zenodo.10203115)^46^.
